# Reduction of Weed Growth under the Influence of Extracts and Metabolites Isolated from *Miconia* spp.

**DOI:** 10.3390/molecules27175356

**Published:** 2022-08-23

**Authors:** Gabriel Rezende Ximenez, Mirelli Bianchin, João Marcos Parolo Carmona, Silvana Maria de Oliveira, Osvaldo Ferrarese-Filho, Lindamir Hernandez Pastorini

**Affiliations:** 1 Programa de Pós-Graduação em Biologia Comparada, Centro de Ciências Biológicas, Departamento de Biologia, Universidade Estadual de Maringá, Avenida Colombo 5790, Maringá 87020-900, Brazil; 2 Programa de Pós-Graduação em Química, Centro de Ciências Exatas, Departamento de Química, Universidade Estadual de Maringá, Avenida Colombo 5790, Maringá 87020-900, Brazil; 3 Graduação em Biotecnologia, Centro de Ciências Biológicas, Departamento de Biotecnologia, Genética e Biologia Celular, Universidade Estadual de Maringá, Avenida Colombo 5790, Maringá 87020-900, Brazil; 4 Programa de Pós-Graduação em Ciências Biológicas, Centro de Ciências Biológicas, Departamento de Biologia Celular e Genética, Universidade Estadual de Maringá, Avenida Colombo 5790, Maringá 87020-900, Brazil

**Keywords:** phytotoxicity, allelochemicals, flavonoids, seedlings, phytochemical analysis, weed control

## Abstract

Weeds pose a problem, infesting areas and imposing competition and harvesting difficulties in agricultural systems. Studies that provide the use of alternative methods for weed control, in order to minimize negative impacts on the environment, have intensified. Native flora represents a source of unexplored metabolites with multiple applications, such as bioherbicides. Therefore, we aimed to carry out a preliminary phytochemical analysis of crude extracts and fractions of *Miconia auricoma* and *M. ligustroides* and to evaluate these and the isolated metabolites phytotoxicity on the growth of the target species. The growth bioassays were conducted with Petri dishes with lettuce, morning glory, and sourgrass seeds incubated in germination chambers. Phytochemical analysis revealed the presence of flavonoids, isolated myricetin, and a mixture of quercetin and myricetin. The results showed that seedling growth was affected in a dose-dependent manner, with the root most affected and the seedlings of the lettuce, morning glory, and sourgrass as the most sensitive species, respectively. Chloroform fractions and myricetin were the most inhibitory bioassays evaluated. The seedlings showed structural changes, such as yellowing, nonexpanded cotyledons, and less branched roots. These results indicate the phytotoxic potential of *Miconia* allelochemicals, since there was the appearance of abnormal seedlings and growth reduction.

## 1. Introduction

Natural plant compounds, produced through secondary metabolism pathways, are called allelochemicals when they are released into the environment and affect the development and growth of neighboring plants or microorganisms. Plants produce and release, in different ways, numerous allelochemicals that affect the development of local biota, such as organic acids and phenolic compounds [1,2]. Flavonoids play important roles in plant development, primarily in modulating the transport of the growth phytohormone auxin [3,4]. Flavonoids can stimulate degradation pathways and affect the stability of membrane transporters, as well as modify plant growth [5,6].

Since it represents a still unexplored source of compounds with diverse biological properties, the study of the chemical potential of plants is necessary to understand the ecophysiological characteristics of species in natural environments and to enable the discovery of molecules with different chemical properties and new applicability (pharmacological, commercial, and agro-industrial, among others) [7].

Two weeds present in several countries are morning glory (*Ipomoea triloba* L.) and sourgrass (*Digitaria insularis* (L.) Fedde, species that are native to America but invasive of many habitats. There are reports that both are resistant to commercial herbicides [8,9] and each are considered very problematic species to control in agricultural cultivation areas [10]. Morning glory has a climbing habit and, as it wraps itself around the plants, it makes harvesting difficult and can affect crop development and yield. Sourgrass, due to its great dissemination potential and tolerance to desiccation, is considered a skillful competitor, occupying the place of cultivated plants and making mechanized harvesting difficult [11]. Thus, the use of alternative methods, such as bioherbicides, has become important to contain the advance of unwanted plants and to minimize environmental impacts by reducing the use of synthetic chemicals [12]. As a result, the interest in the research on natural herbicides has grown in recent years, since many plants produce and store different natural compounds with numerous properties to be explored [13,14]. Compared to other countries, Brazil has not yet registered and marketed such products; however, the country stands out in research in search of potential bioherbicides [15].

One of the aspects that attract researchers in search of metabolites with biological properties is the wide diversity of the Brazilian territory, and thus the great diversity of the national flora, since native species are the sources of new bioactive compounds. Campos Gerais is one of these regions, located in the state of Paraná, and has highly diverse vegetation fragments [16]. Considering the floristic formations, it is a zone where patches of Cerrado occur [17] in humid tropical and subtropical forests [18].

The Brazilian Cerrado is considered the habitat with the greatest biodiversity on the planet, with a high rate of species endemism. It suffers greatly from degradation and is at risk of losing its biodiversity [19]. Amidst the patches of Cerrado in Paraná is located the Guartelá State Park (PEG), with a very diverse vegetation which extensively covers the park, varying between different forest patches, rocky outcrops, and clean fields [20,21].

Among the groups of plants characteristic of this region is the family Melastomataceae A.Juss. According to the research [22], this family includes about 1340 species of Brazilian flora, grouped into 69 genera, common in all phytogeographic domains but with greater richness in the Atlantic Forest, Cerrado, and the Amazon [23]. In this family, the genus *Miconia* stands out. Studies on the chemical composition of the genus *Miconia* showed that the main metabolites found in the group are flavonoids and terpenes. Biological evaluations of these compounds have shown several pharmacological and medicinal properties [24]. However, recent studies have focused not only on the medicinal but also on the allelochemical applicability of these secondary metabolites. Few studies have reported the phytotoxic and allelopathic action of the extracts, and even fewer have reported on the compounds isolated from species belonging to this genus [25,26,27,28,29,30,31,32,33,34].

Thus, this study aims to carry out a preliminary phytochemical analysis of the crude extracts and chloroform fraction obtained from the aerial parts of *Miconia auricoma* and *Miconia ligustroides* and to evaluate these and the isolated metabolites’ phytotoxicity through initial growth bioassays with the seedlings of the weed species morning glory and sourgrass, as well as the indicator, lettuce.

## 2. Results

### 2.1. Initial Growth Bioassays with CE and FCHCl_3_ of Miconia sp.

**Figure 1:**
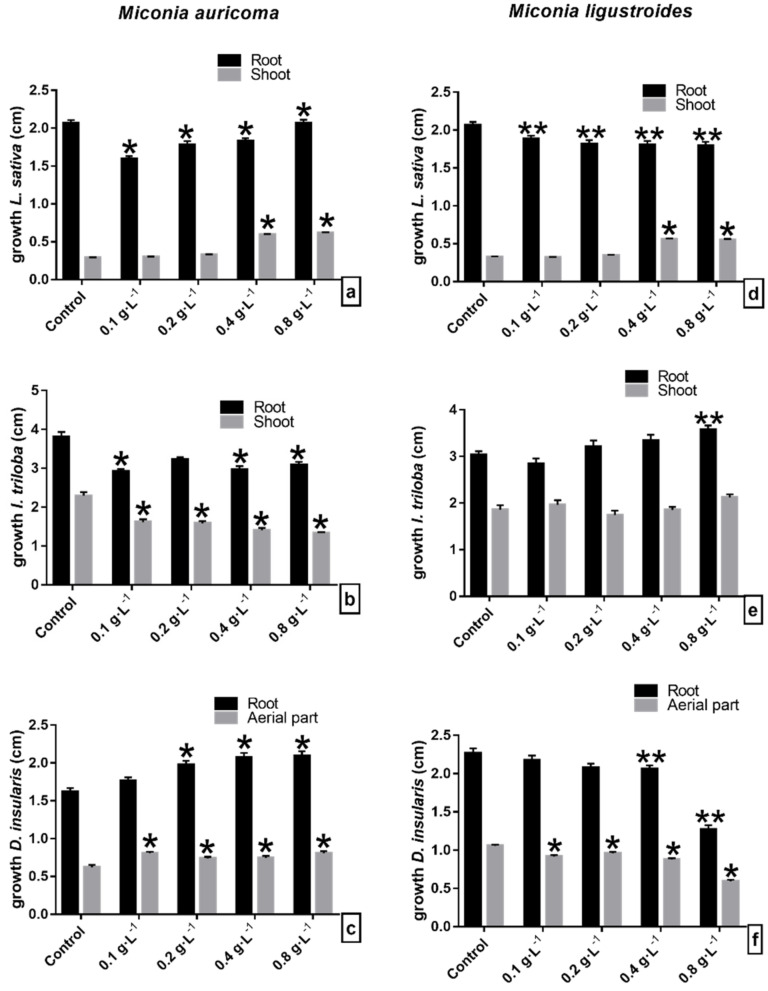
Initial growth of seedlings of (**a**–**d**) lettuce (*L. sativa*), (**b**–**e**) morning glory (*I. triloba*), and (**c**–**f**) sourgrass (*D. insularis*) under crude extract of (**a**–**c**) *Miconia auricoma* and (**d**–**f**) *Miconia ligustroides*. ** Indicates means ± SEM with statistically significant differences by Dunnett’s test (*p* < 0.05) compared to the test control; * Indicates means ± SEM with statistically significant differences by Dunn’s test (*p* < 0.05) compared to the control test.

**Figure 2:**
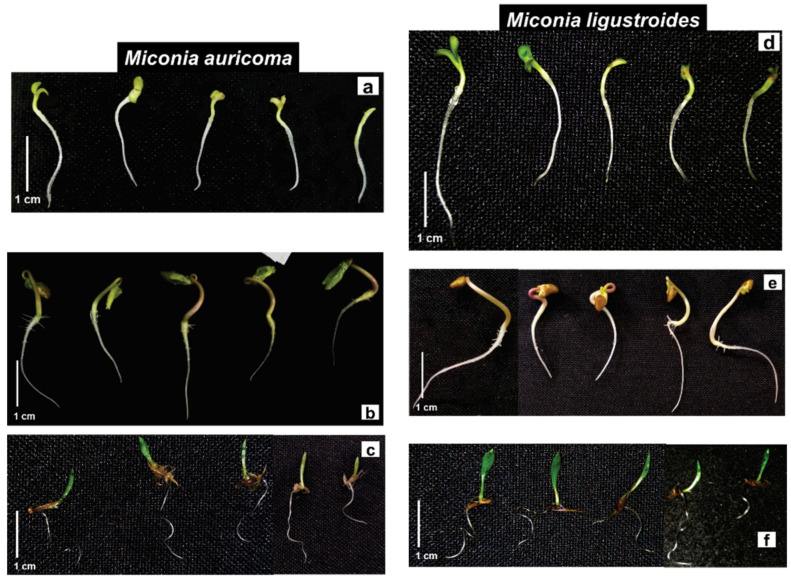
Morphological aspect of (**a**–**d**) lettuce (*L. sativa*), (**c**–**e**) morning glory (*I. triloba*), and (**c**–**f**) sourgrass (*D. insularis*) seedlings under crude extract (CE) of *Miconia auricoma* and *Miconia ligustroides* (left to right: control; 0.1, 0.2, 0.4, and 0.8 g·L^−1^; scale 1 cm).

Lettuce seedling growth was susceptible to crude extract (CE) allelochemicals from *Miconia* sp. Under *M. auricoma*, the root length was inhibited, as it significantly reduced the root growth inversely (the more concentrated the solution, the greater the root elongation) (Figure 1a). Under the CE of *M. ligustroides*, lettuce roots were affected according to the concentration. Root elongation was significantly inhibited between 8 and 13% (Figure 1d). However, both the CEs of *Miconia* sp., at the highest concentrations, stimulated the length of the shoot. Under *M. auricoma*, the growth of the shoot was significantly stimulated from 4 to 113% by concentration, and the largest hypocotyls were recorded at concentrations between 0.2 and 0.4 g·L^−1^ (Figure 1a), and similar occurred under *M. ligustroides*, with significant stimuli around 7% (Figure 1d). Abnormal lettuce seedlings were observed in the bioassays. The highest concentrations caused the presence of yellowish seedlings, with nonexpanded cotyledon ends and signs of blackening (Figure 2a,d).

In the morning glory seedlings, *M. auricoma* CE significantly reduced the length of the shoot at concentrations of 0.1, 0.4, and 0.8 g·L^−1^ at around 21% compared to the control (Figure 1b), differently from what happened with the *M. ligustroides* CE, since between the concentrations of 0.1 and 0.4 g·L^−1^, root growth did not differ from that of the control; under the concentration of 0.8 g·L^−1^, on the other hand, a stimulus to the root growth was observed (Figure 1e). The growth of the shoot was significantly inhibited with increasing concentrations of *M. auricoma* CE in proportions of 29–41% (Figure 1b). Seedlings treated with the *M. ligustroides* CE showed significant reductions or stimulation of aerial growth when compared with the control (Figure 1e). The appearance of seedlings with less ramification in the root/hypocotyl transition area, twisted hypocotyls, and with cotyledonary leaves contained in the seminal tegument and with a yellowish color was a common finding (Figure 2b,e).

Sourgrass seedlings were stimulated when treated with the *M. auricoma* CE solutions. The root grew around 9–29% gradually with increasing concentrations, statistically different from the assay control of 0.2 g·L^−1^ (Figure 1c). However, under the *M. ligustroides* CE, root elongation was inhibited, so that the concentration of 0.8 g·L^−1^ was the most effective and significantly reduced the root growth of the sourgrass by 44%. Likewise, a 43% reduction in the aerial growth was observed, statistically different from the bioassay control (Figure 1f). Under the *M. auricoma* CE, the aerial part was significantly stimulated at the concentrations evaluated (Figure 1c). As for the morphological aspect, the seedlings presented a curled eophyll limb with a less expanded aspect, mainly under the highest concentrations (Figure 2c,f).

**Figure 3:**
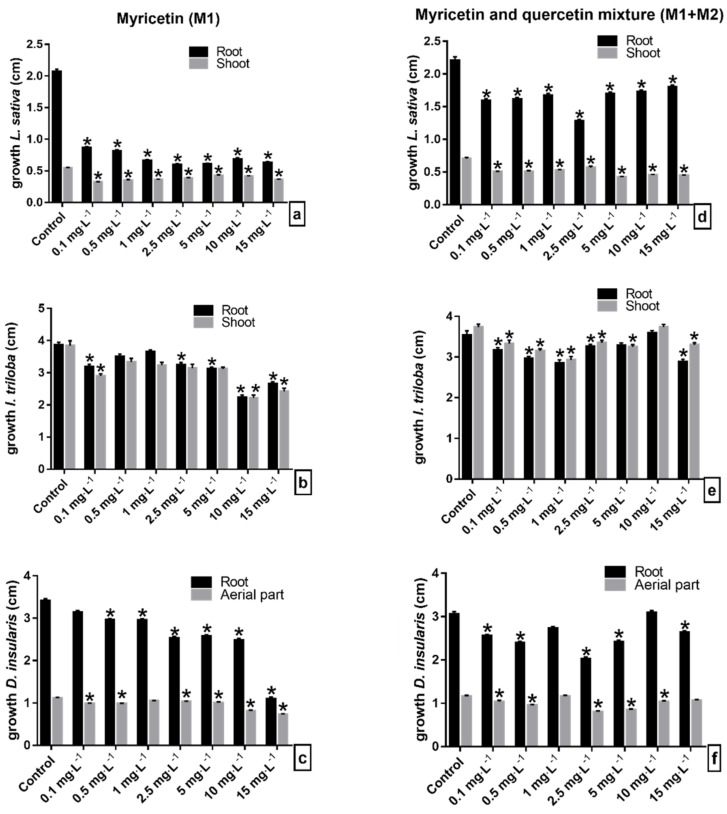
Initial growth of seedlings of (**a**–**d**) lettuce (*L. sativa*), (**b**–**e**) morning glory (*I. triloba*), and (**c**–**f**) sourgrass (*D. insularis*) under chloroform fraction of (**a**–**c**) *Miconia auricoma* and (**d**–**f**) *Miconia ligustroides*: (**a**–**d**) lettuce, (**b**–**e**) morning glory, and (**c**–**f**) sourgrass. * Indicates means ± SEM with statistically significant differences by Dunn’s test (*p* < 0.05) compared to the control test.

**Figure 4:**
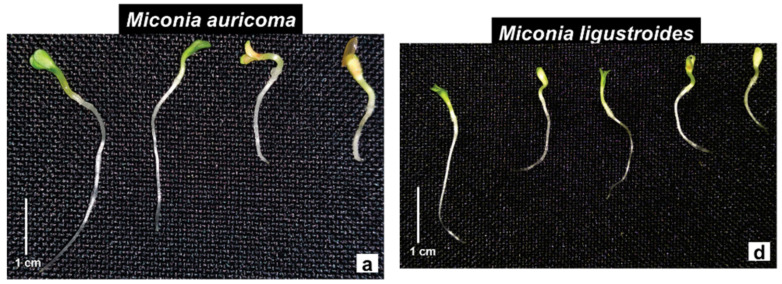

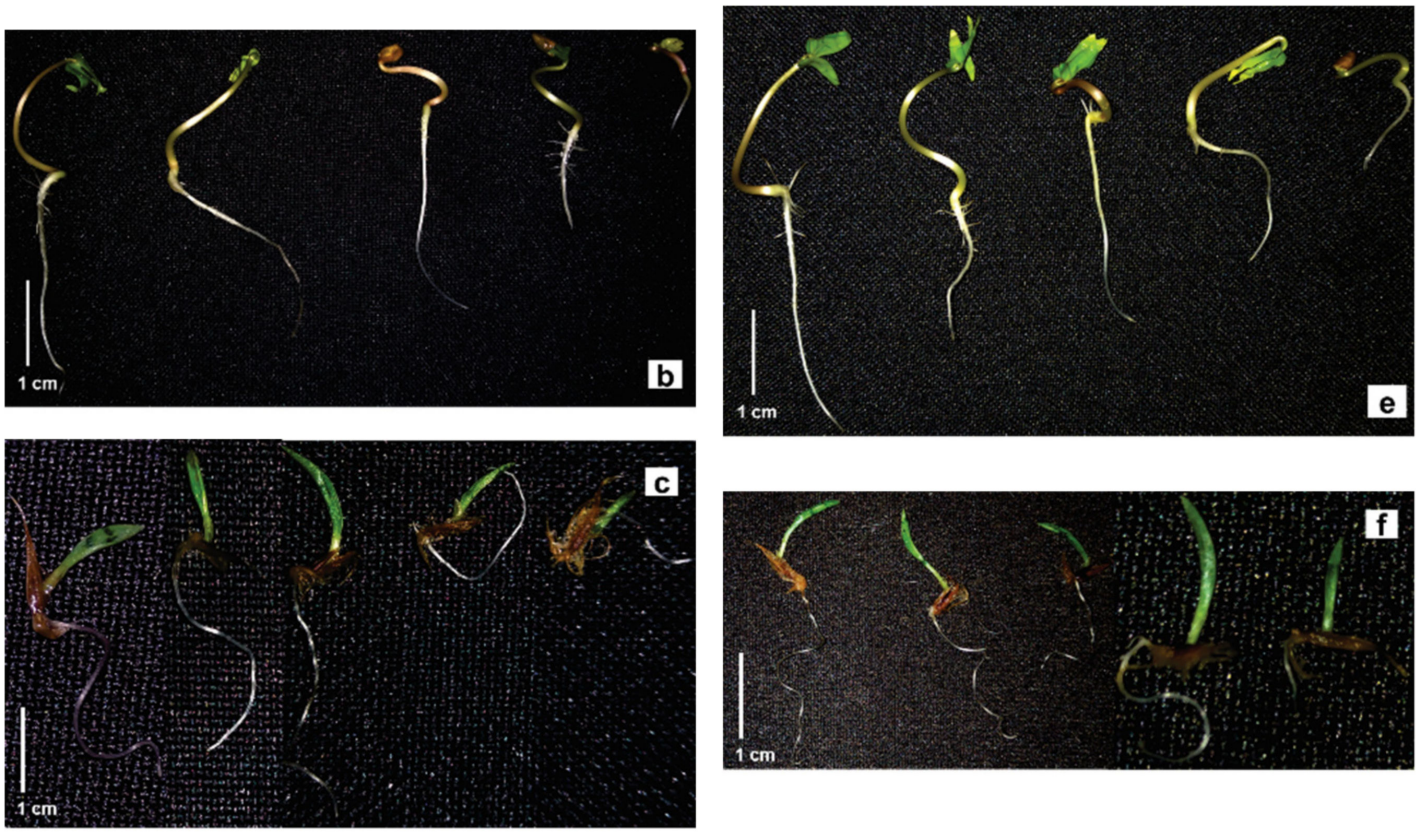
Morphological aspect of (**a**–**d**) lettuce (*L. sativa*), (**b**–**e**) morning glory (*I. triloba*), and (**c**–**f**) sourgrass (*D. insularis*) seedlings under chloroform fraction (FCHCl_3_) of *Miconia auricoma* and *Miconia ligustroides* (left to right: control; 0.1, 0.2, 0.4, and 0.8 g·L^−1^; scale 1 cm).

Under the *M. auricoma* chloroform fraction (FCHCl_3_) solutions, the root growth of the lettuce seedlings was significantly reduced with increasing concentrations from 0.2 g·L^−1^. Seedlings did not grow under the concentration of 0.8 g·L^−1^ (Figure 3a). Significant root inhibitions also occurred in bioassays with *M. ligustroides* FCHCl_3_, except at the 0.2 g·L^−1^ concentration (Figure 3d). The shoot elongation of the lettuce seedlings was significantly reduced by 10% at a 0.1 g·L^−1^ concentration and stimulated by 16% at a 0.2 g·L^−1^ concentration with the *M. auricoma* FCHCl_3_ solutions (Figure 3a). The same occurred with the seedlings under *M. ligustroides* FCHCl_3_, in which the aerial length of the seedlings was between 12% and 16%, significantly higher than the bioassay control at concentrations of 0.2 and 0.4 g·L^−1^. However, growth was reduced by 13% and was statistically different from the control under the concentration of 0.1 g·L^−1^ (Figure 3d). Lettuce seedlings under FCHCl_3_ showed yellowing, unexpanded cotyledons, and blackening of the root apex under the highest concentrations (Figure 4a,d).

Morning glory seedlings were susceptible to the phytotoxic effects of *M. auricoma* FCHCl_3_. Root growth was progressively inhibited with increasing concentrations, with a mean reduction of 28–65%, and was statistically different from the control (Figure 3b). The same occurred in bioassays with *M. ligustroides* FCHCl_3_, with significant average inhibitions of 40% (Figure 3e). *M. auricoma* FCHCl_3_ significantly reduced the length of the shoot, except at a 0.4 g·L^−1^ concentration (Figure 3b). *M. ligustroides* FCHCl_3_ reduced the length of the shoot by an average of 29% at all concentrations tested, and it was statistically different from the control (Figure 3e). Similar observations occurred with the seedlings under the *M. ligustroides* FCHCl_3_ solutions. Morning glory seedlings with twisted hypocotyls, cotyledonary leaves with yellowish ends, and which were not expanded or contained in the seminal tegument were found in the bioassays with *Miconia* sp. FCHCl_3_ (Figure 4b,e).

Sourgrass seedlings under *M. auricoma* FCHCl_3_ had significantly reduced root growth, so that the concentrations of 0.4 and 0.8 g·L^−1^ were the most inhibitory, with mean reductions of 42% in the root length (Figure 3c). The concentrations of *M. ligustroides* FCHCl_3_ gradually inhibited the roots, with increasing concentrations with statistical differences from 0.2 g·L^−1^ and an average reduction of 63% in the most concentrated solutions (Figure 3f). The length of the sourgrass aerial parts seedlings was significantly inhibited, around 18% under *M. auricoma* FCHCl_3_ concentrations (Figure 3c). *M. ligustroides* FCHCl_3_ inhibited the aerial growth at concentrations of 0.4 and 0.8 g·L^−1^, with a mean reduction of 37% (Figure 3f). Sourgrass seedlings treated with *Miconia* FCHCl_3_ showed curling in the eophyll limb, decreased leaf area, and less expansion in the elongation (Figure 4c,f).

### 2.2. Initial Growth Bioassays with Flavonoids Myricetin (M1) and Myricetin + Quercetin Mixture (M1 + M2) from M. ligustroides

**Figure 5:**
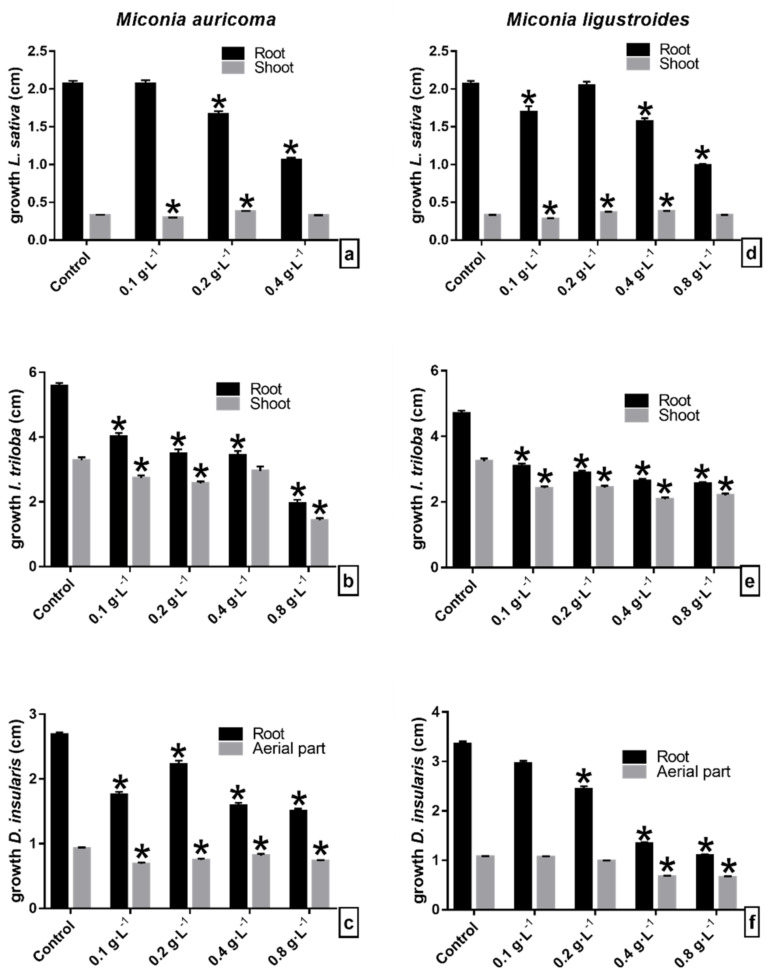
Initial growth of seedlings of (**a**–**d**) lettuce (*L. sativa*), (**b**–**e**) morning glory (*I. triloba*), and (**c**–**f**) sourgrass (*D. insularis*) under solution of flavonoids (**a**–**c**) myricetin (M1) and (**d**–**f**) myricetin+quercetin mixture (M1 + M2). * Indicates means ± SEM with statistically significant differences by Dunn’s test (*p* < 0.05) compared to the control test.

**Figure 6:**
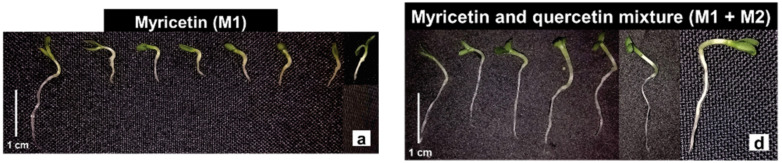

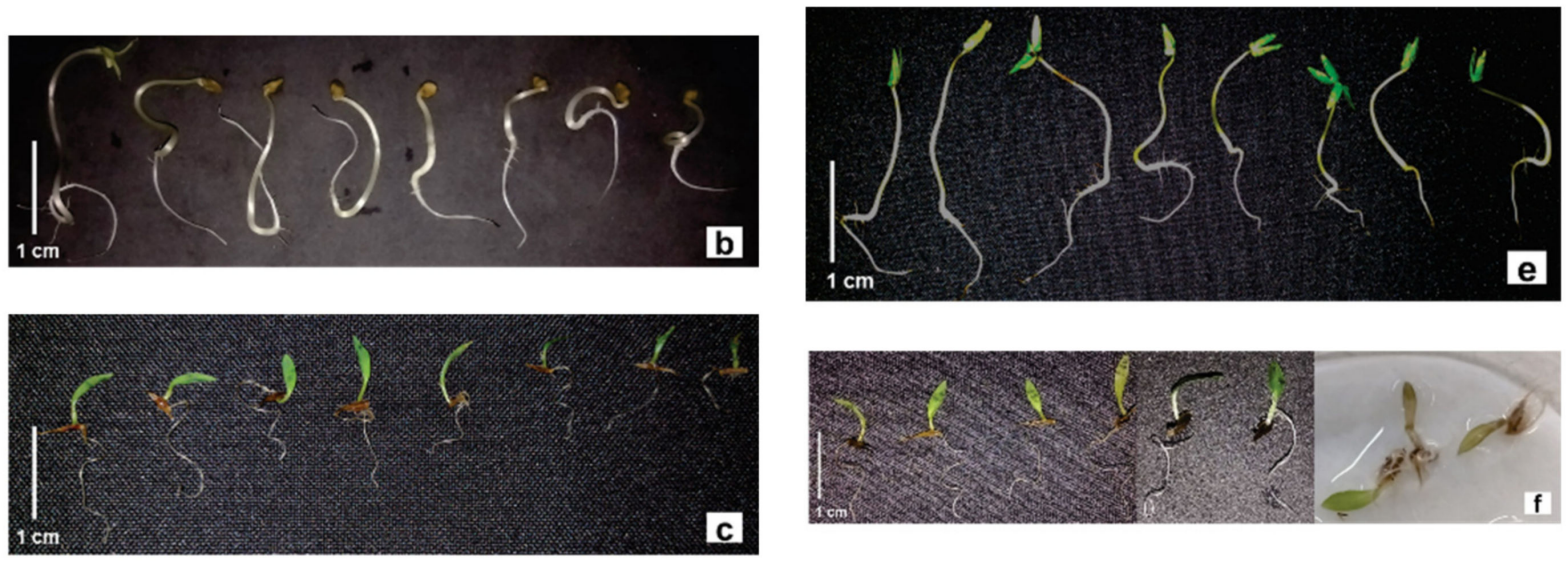
Morphological aspect of (**a**–**d**) lettuce (*L. sativa*), (**b**–**e**) morning glory (*I. triloba*), and (**c**–**f**) sourgrass (*D. insularis*) seedlings under (**a**–**c**) myricetin (M1) and (**d**–**f**) myricetin + quercetin mixture (M1 + M2) (left to right: control; 0.1, 0.5, 1, 2.5, 5, 10, and 15 g·L^−1^; scale 1 cm).

The flavonoids myricetin (M1) and the myricetin+quercetin mixture (M1 + M2) showed phytotoxic effects on the seedlings of the species tested. Lettuce seedlings under the M1 solutions showed a significant reduction in root growth by an average of 66% compared to the control, and the smallest sizes measured were recorded under the concentration of 2.5 mg·L^−1^ and 5 mg·L^−1^ (Figure 5a). Similar results were obtained in the bioassays with the flavonoid mixture M1 + M2, in which the concentrations evaluated inhibited an average of 26% of the root length (Figure 5d). As in the root, the shoot was significantly inhibited in the evaluated concentrations of the flavonoids in comparison to the control, so that the concentrations of 0.1 and 5 mg·L^−1^ reduced the shoot growth by 40%, under the solutions of the flavonoids M1 and the M1 + M2 mixture, respectively (Figure 5a,d). Under the M1 solutions, the lettuce seedlings showed a delay in the expansion and opening of the cotyledons and a slight darkening of the root in seedlings under the highest concentrations (Figure 6a). In contact with the M1 + M2 mixture, the occurrence of seedlings with elongated and tapered roots and a translucent appearance was common (Figure 6d).

Morning glory seedlings were susceptible to the phytotoxic effects of flavonoids at a concentration of 0.1 mg·L^−1^. At concentrations from 2.5 mg·L^−1^ of M1, the root growth was significantly reduced, with a mean reduction of 27%. Similar results occurred when the seedlings were treated with the M1 + M2 mixture, except at a concentration of 10 mg·L^−1^ (Figure 5b,e). However, the other concentrations of the M1 + M2 mixture significantly reduced the root of the morning glory seedlings by around 13% (Figure 5e). Under the M1 concentrations, the shoot of the morning glory was significantly reduced, so that the concentrations 10 and 15 mg·L^−1^ were the ones that most reduced the elongation of the structure, followed by 0.1 mg·L^−1^, and they inhibited it by 42% and 37%, respectively (Figure 5b). Under the M1 + M2 mixture, except at 10 mg·L^−1^, the growth was significantly reduced by an average of 14% (Figure 5e). Under M1, the morning glory seedlings showed abnormal growth, such as the discoloration of the shoot, roots with signs of atrophy and warping, decreased branching, twisted hypocotyls, and cotyledons contained in the seminal tegument (Figure 6b). Morning glory in contact with the M1 + M2 mixture showed reduced branching of the roots, yellowing, and reduced opening of cotyledonary leaves and slight torsions of the hypocotyl (Figure 6e).

The M1 solutions significantly reduced the root growth of the sourgrass seedlings with increasing concentrations from 0.5 mg·L^−1^ (Figure 5c). Significant reduction in root growth also occurred in seedlings treated with the M1 + M2 mixture, except at the 10 mg·L^−1^ concentration (Figure 5f). The aerial growth of the sourgrass seedlings was significantly inhibited under the M1 and M1 + M2 mixture solutions, except at the concentrations 1.0 and 15 mg·L^−1^, respectively (Figure 5c,f). Sourgrass seedlings presented different morphological appearances under the concentrations of the evaluated flavonoids: eophylls with curled, yellowish appearance, not expanded and occasionally blackened, and some seedlings with shorter, stunted roots (Figure 6f).

## 3. Discussion

**Figure 7:**
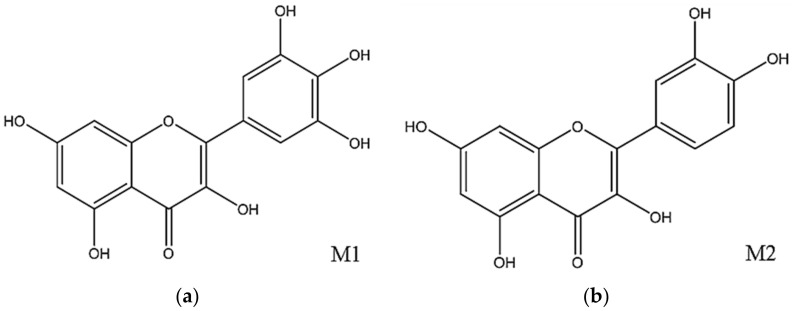
Molecular structure of the flavonoids: (**a**) Myricetin (M1) and (**b**) Quercetin (M2).

Preliminary phytochemical analysis of the *M. ligustroides* extracts revealed the presence of two secondary metabolites belonging to the flavonoid group, myricetin (M1) and myricetin mixed with quercetin (M2) (Figure 7a,b). Thus, the allelochemical activity of *M. ligustroides* CE, FCHCl_3_, and isolated flavonoids can be ascribed to the effects of these constituents in affecting the development of the analyzed species, since the flavonoids can negatively influence the regulation of auxin, the main hormone of plant growth. Preliminary phytochemical analysis of *M. auricoma* revealed the majority presence of ursane, oleanane, and di- and trihydroxylated triterpenes [35]. However, this does not rule out the presence of other classes of compounds in the material used. Thus, the results found in the bioassays can be attributed to these constituent metabolites and, eventually, to others to be identified.

The results show that the growth of the evaluated seedlings was significantly affected by the allelochemicals present in the *Miconia* CE and FCHCl_3_. The seedling root was more sensitive than the shoot to the phytotoxic effects of the constituent compounds. Furthermore, the bioassay solutions, in general, inhibited the root growth in a dose-dependent manner; that is, as the evaluated concentration increased, the growth of the organ decreased proportionally (Figure 1 and Figure 3). The *Miconia* FCHCl_3_ showed greater phytotoxic effects on root growth, and lettuce was the most sensitive species (Figure 3). Morning glory roots also showed greater sensitivity to the phytotoxic effects of the fraction as compared to the sourgrass roots. However, this marked inhibition of root growth was not observed in the shoot of the lettuce seedlings. The results showed that the solutions gradually stimulated the aerial elongation in relation to the increase in the concentration of *Miconia* CE and FCHCl_3_, suggesting that the lettuce shoot was less sensitive than the root to *Miconia* metabolites. This difference in organ sensitivity may be related to the modified allelochemical response, as the responsiveness depends on the substance, the concentration being tested, and the target species evaluated [36,37], together with the fact that the roots are in direct contact with the allelochemicals of the solutions used in the bioassays. Similar results were obtained when testing the aqueous extracts of *Miconia* spp. on the lettuce growth, reporting that there was no effect on the aerial growth; however, in particular, *M. ligustroides* showed phytotoxicity and altered the cell division of root cells and inhibited root growth [34]. Other studies observed similar results to the performed bioassays, since *M. coronata* and *M. calaletti* fractions were more phytotoxic in the root than in the shoot growth of the lettuce seedlings [27,28] and *M. coronata* and *M. aeruginosa* extracts in tomato seedlings [26]. Therefore, the occurrence of phytotoxic effects, as shown in the tests with CE and fractions, can be attributed to the metabolites (flavonoids and the others not identified) present in the material used in the bioassay solutions.

Regarding the bioassays with the flavonoids myricetin (M1) and the myricetin + quercetin mixture (M1 + M2), the lettuce seedlings were the most sensitive to these compounds, followed by the morning glory and sourgrass seedlings (Figure 5). The smallest lettuce seedlings were obtained at a concentration of 2.5 mg·L^−1^ of the compounds of the M1 and M1 + M2 mixture, while the most evident reduction in the size of the morning glory and sourgrass seedlings kept in the solution with the compound M1 occurred at concentrations of 10 mg·L^−1^ and 15 mg·L^−1^, respectively. This corroborates with the previously mentioned reports in the literature, as the allelochemical effect can be differentiated in relation to the concentration of the substance, since the different concentrations of the compounds presented different rates of inhibition in the species evaluated in the bioassays.

Flavonoids compose the most abundant class of phenolic compounds and have numerous pharmacological activities, such as antioxidant, dietary, anti-inflammatory, antimicrobial, antitumor, antilipemic, and cytoprotective activity, among others [38,39,40]. In plant development, flavonoids can stimulate the auxin (growth hormone) oxidation pathway and affect its transporters, and thus modulate plant growth [41]. According to the research, the flavonoids identified in the FAcOEt of *M. ligustroides*, myricetin (M1), and quercetin (M2) are the most abundant in plants and have antioxidant action [42,43]. Many studies report the allelochemical activity of quercetin, whereas information about myricetin is scarce. Most of the studies are of a pharmacological nature.

Myricetin and quercetin, besides being antioxidants, act in defense and protect plant roots against parasitic nematodes [44]. In contrast to its activity as an antioxidant, myricetin can act as an oxidant of Fe^2+^ ions and thus can be harmful to the DNA structure [45], and at the same time can act as a mutagenic and cytoprotective substance to animal cellular DNA [46,47], a role that is related to its molecular structure. In this way, myricetin can also be harmful to plant cells, since, as it exhibits genotoxic properties, as described above, it can affect the cell division process of plant tissues, organelles such as mitochondria and chloroplasts, and affect the homeostatic balance and the photosynthetic apparatus through oxidative stress, and thus inhibit seedling growth.

Studies have reported that quercetin is a potent inhibitor of the auxin transport [6,41,48]. When accumulating in the meristematic zone of the root apex, quercetin affects the transport of the hormone to adjacent cells and alters the auxin gradient, which is necessary for the elongation of the organ’s cells, which delays growth [5,49,50]. Auxin is a key phytohormone in all major plant development processes and acts directly on cell division, elongation, and differentiation. When its activity is affected, it can thus modify tissue formation and architecture and alter seedling morphogenesis [51,52]. Thus, with the results obtained in the bioassays with these flavonoids, we propose that both affected one or more of the processes described above, and thus inhibited the growth of the evaluated species, through the reduction in the development of the root and shoot in the seedlings, in particular the severe inhibition obtained in the growth of lettuce seedlings under myricetin solutions.

Studies report that quercetin inhibited the root growth of *Arabidopsis thaliana* [53], similar to what was also observed when analyzing the root growth of sicklepod (*Senna obtisuifolia*) [54] and on the growth of morning glory [55]. Quercetin caused a decrease in the respiration in soybean mitochondria [56]. In studies with buckwheat (*Fagopyrum esculentum*), quercetin and myricetin was obtained, which in bioassays showed that only quercetin reduced the growth of weeds (*Echinochloa crus-galli*, *Lolium perenne*, *Sinapis alba*, and *Trifolium repens*), whereas myricetin was not effective [57]. However, the report showed that myricetin and quercetin inhibited the aerial and root growth of lettuce seedlings [58]. These data corroborate the results obtained in the performed bioassays, since myricetin was effective in reducing the growth of the evaluated seedlings, especially lettuce.

The seedling is a stage of plant development that is very sensitive to biotic and abiotic factors in the environment [59]. Through the immediate contact with the metabolites of the solutions, the roots are greatly affected by the phytotoxicity of these constituents; thus, they can be the main target of the effects of secondary metabolites. Reduction in organ growth and the appearance of morphological abnormalities are typical consequences of allelochemical inhibition [10,60]. These observations agree with the results, since the bioassays caused the appearance of seedlings with morphological changes. A decrease in the seedling growth was observed, probably due to the organ growth deficit (reduction in shoot and root growth), and therefore reducing seedling establishment [61], and also decreasing the photosynthetic rate, because, as reported, the cotyledon structure was damaged (delayed expansion and yellowing). Twisted hypocotyls, nonexpanded, yellowish cotyledonary leaves, still contained in the seminal tegument, delayed opening and expansion of the eophyll and roots with little or no ramifications, with signs of blackening and atrophy were common in the bioassays (Figure 2, Figure 4 and Figure 6).

Other studies also reported similar results, in which flavonoid-rich fractions of aerial parts of Fabaceae caused morphological changes in lettuce, wild-poinsettia (*Euphorbia heterophylla*), morning glory, and sourgrass [62,63]. Changes in branching and root hair formation, similar to the results shown here, were also reported in red rice and barnyard grass seedlings grown with extracts and constituents of *Tinospora tuberculata* [64] and on morning glory and sourgrass seedlings [65]. Extracts of sicklepod (*Senna occidentalis*) caused blackening in the roots, altered root branching, shoot dwarfism, and discoloration in *Tabebuia* seedlings [66]. Allelochemicals decrease seedling growth as a secondary response. First, they affect cellular structures directly involved in metabolism, such as organelles, or modify the balance of hormones linked to morphogenesis, such as auxin. These modifications can cause changes in cellular respiration, photosynthetic reactions and redox reactions and consequently decrease seedling growth by affecting cellular homeostasis [67,68]. This decrease is often related to the drop in root cell viability [69]. The darkening and the occurrence of necrosis are directly related to the loss of viability of cells that are part of the organ. Roots damaged or malformed by the phytotoxicity of allelochemicals will affect plant growth and development and cause the occurrence of plants with an abnormal appearance [70]. These results clearly show that the root is the organ most affected by allelochemicals in phytotoxic assays and the changes that occur in the evaluated species are signs of the allelochemical influence. These findings corroborate the results of our bioassays with the CE, fractions, and compounds evaluated.

The flavonoids myricetin and quercetin from *M. ligustroides* were present not only in the FAcOEt, but also in the CE, since the fraction was obtained after the liquid:liquid partition of the CE. Therefore, based on the results shown, it is possible to propose that the phytotoxic effects found in the bioassays with the *M. ligustroides* CE solutions can be attributed to the presence of these allelochemicals, since the CE used is not partitioned material. However, in FCHCl_3_, other constituent metabolites may be present, different from those isolated in polar solvents, given that chloroform has apolar characteristics in relation to ethyl acetate [71]. In addition, the phytochemical analysis techniques used were not successful in identifying and/or isolating constituents other than those described above. The same can be said for the CE and FCHCl_3_ of *M. auricoma*, which indicated the presence of triterpenes as major compounds despite not having isolated constituents. Every plant organ produces secondary metabolites in different concentrations and classes, so it can be said that the compounds present in the material used had allelochemical properties and affected the development of the evaluated seedlings.

## 4. Materials and Methods

### 4.1. Plant Material

The species *Miconia ligustroides* and *Miconia auricoma* were collected, respectively, on 13–14 September 2017, at Parque Estadual do Guartelá (PEG) in Tibagi-PR, under the geographic coordinates: latitude: −24.33411 and longitude: −50.15199 WGS84, and latitude: −24.33665 and longitude: −50.1528 WGS84. The exsiccates were deposited in the Herbarium of Universidade Estadual de Maringá (HUEM) under the code HUEM 33157 for *M. ligustroides* and HUEM 33154 for *M. auricoma*.

After collection, the aerial parts of *M. ligustroides* and the flowers of *M. auricoma* were dried at room temperature and ground in a knife mill. For extraction, 980 g of aerial parts of *M. ligustroides* and 150 g of flowers of *M. auricoma* were subjected to exhaustive maceration in cold methanol (12 times of 1 L for *M. ligustroides* and 9 times of 1 L for *M. auricoma*). The extracts were concentrated in a rotary evaporator at 37 °C and yielded 47.7 g of gross extract of aerial parts of *M. ligustroides* (MLEB) and 41.85 g of extract of flowers of *M. auricoma* (LAEB).

### 4.2. Fractionation of Plant Extracts

Part of the extracts, 32.27 g of MLEB and 37.30 g of LAEB, were subjected to liquid:liquid partition with solvents of different polarities (hexane, chloroform, and ethyl acetate). Initially the extracts were solubilized in a mixture of MeOH/H_2_O 1:1, followed by exhaustive extractions with hexane. The remaining methanol–water solutions were concentrated to volume reduction, followed by the addition of water. Subsequently, partitioning was carried out until exhaustion with chloroform and ethyl acetate. In this process, the hexane (MLHex and LAHex), chloroform (MLCHl_3_ and LACHl_3_), ethyl acetate (MLAcOEt and LAAcOEt) fractions, the hydromethanolic remnant (MLHM and LAHM) for aerial parts of *M. ligustroides*, and the flowers of *M. auricoma*, respectively, were obtained. The volumes of each solvent used, and the masses obtained for each fraction, are described in Table 1.

### 4.3. Isolation and Characterization of Substances

To obtain the flavonoid myricetin (M1) (Figure 7a), part of the ethyl acetate fraction (4.45 g) was subjected to extraction using the direct contact method of the sample with solvents of different polarities. Initially, 100 mL of dichloromethane was added to the fraction, followed by stirring and separating the soluble part. To the remaining fraction, 100 mL of ethyl acetate was added, followed by methanol (100 mL) and water (100 mL), and the dichloromethane (MLAcOEt-1), ethyl acetate (MLAcOEt-2), methanolic (MLAcOEt-3), and aqueous (MLAcOEt-4) subfractions were obtained.

The MLAcOEt-3 subfraction (1.02 g) was subjected to chromatography on a Sephadex LH-20 eluted with 100% MeOH (Ø = 1.6 cm, h = 47 cm), which resulted in 18 subfractions, grouped based on TLC (thin layer chromatography) analysis. Subfraction 14 resulted in the isolation of the substance myricetin (M1) (Figure 7a), which was identified through 1H and 13C NMR spectroscopic data and according to literature reports [72].

From the study of the hydromethanolic fraction (MLHM), the substance myricetin (M1) (Figure 7a) was also isolated but mixed with quercetin (M2) (Figure 7b). For this, part of MLHM (10.56 g) was solubilized in methanol (40 mL), followed by the addition of ethyl ether (60 mL) and centrifugation (10 min at 3000 rpm, 25 °C) for precipitation and tannins removal. This process was repeated three times to obtain the tannic subfraction (MLHM-1T) and the tannin-free fraction (MLHM-2).

The MLHM-2 subfraction (4.65 g) was subjected to a sephadex LH-20 chromatographic column eluted with MeOH/H2 O 1:1 (Ø = 1.6 cm, h = 32.0 cm), obtaining 14 subfractions assembled based on the TLC profile. Subfraction 13 resulted in the isolation of the mixture of myricetin substances (Figure 7a), and through 1H and 13C NMR spectroscopic data, the substance M2 was identified as quercetin (Figure 7b) [73]. By means of 1H and 13 CNMR spectroscopic data, it was possible to identify the substances and establish the proportion of each one in the mixture: M1: myricetin 79.4% + M2: quercetin 20.6%.

### 4.4. Preparation of Solutions of CE and FCHCl_3_ Fraction

Aliquots of 40 mg of the CE and FCHCl_3_ fraction of *M. auricoma* and *M. ligustroides* were solubilized by adding 40 µL of chloroform and 40 µL of methanol and diluted with distilled water to 50 mL (*m*/*v*). From this volume, a 25 mL aliquot was taken, and the remaining 25 mL was diluted again with distilled water. This dilution was repeated four times to obtain concentrations of 0.8 g·L^−1^, 0.4 g·L^−1^, 0.2 g·L^−1^, and 0.1 g·L^−1^.

### 4.5. Preparation of Flavonoids Stock Solutions

The stock solution was prepared by solubilizing 3 mg of the compounds myricetin (M1) and the myricetin + quercetin mixture (M1 + M2) with the addition of 80 µL of methanol, with the subsequent dilution in 150 mL of distilled water. From the stock solution, dilutions were made to obtain concentrations of 15.0, 10.0, 5.0, 2.5, 1.0, 0.5, and 0.1 mg·L^−1^.

### 4.6. Initial Growth Bioassays

The weeds morning glory (*Ipomoea triloba* L.) and sourgrass (*Digitaria insularis* (L.) Fedde) were acquired from the company Agro Cosmos and the vegetable lettuce (*Lactuca sativa* L. cv. Great lakes 659) from a local market.

To overcome dormancy, the morning glory seeds were submerged in H_2_SO_4_ P.A. for 40 min, followed by washing in running water for 5 min [74,75]. Morning glory seeds, sourgrass caryopses, and lettuce cypselas were placed to germinate in a germination chamber under a 12 h photoperiod (light–dark), and for 24 h for morning glory and lettuce, at temperatures of 30 °C and 25 °C, respectively, and for 48 h at 30 °C for sourgrass caryopses.

After radicle protrusion, 15 seedlings were transferred to Petri dishes with two filter paper disks and irrigated with the solutions. Each treatment consisted of eight replicates (n = 8), and plates with only distilled water with 40 µL of chloroform and 40 µL of methanol used in the solubilization of the 40 mg aliquots were used as controls.

The plates were incubated for 48 h and 72 h in a germination chamber under a 12 h photoperiod (light–dark) at 30 °C for the morning glory and sourgrass weeds, respectively, and under the temperature of 25 °C for 48 h for lettuce. To evaluate the growth, five seedlings of each plate (40 in all) had the length (cm) of the shoot and of the main root measured with millimeter paper.

### 4.7. Statistical Analysis

The experimental design was completely randomized, and the results obtained were evaluated with ANOVA followed by Dunnett’s multiple comparison test (*p* < 0.05). When the assumptions of the parametric analysis were not met, the data were analyzed using the Kruskal–Wallis test (nonparametric ANOVA), followed by Dunn’s test of multiple comparisons (*p* < 0.05). GraphPad Prism^®^ (version 7.0, GraphPad Software Inc., San Diego, CA, USA) was used for the estimating. The results of the bioassays are expressed as the mean ± standard error of the mean (SEM) in column graphs compared to the control of the bioassays with the evaluated conditions that present a statistical difference at 5% of significance.

## 5. Conclusions

This study showed that the crude extracts and fractions of *Miconia auricoma* and *M. ligustroides* and their isolated metabolites negatively affected the growth of lettuce seedlings and of the morning glory and sourgrass weeds. Bioassays showed that seedling growth was affected in a dose-dependent manner, and the root was the most affected organ.

The chloroform fractions and the isolated flavonoid myricetin caused the greatest phytotoxic effect on the evaluated seedlings. The appearance of the seedlings with morphological changes such as yellowing and nonexpanded cotyledons and less branched roots was common.

The results indicate the phytotoxic potential of *Miconia* allelochemicals due to the appearance of abnormal seedlings and the reduction in growth.

## Figures and Tables

**Table 1:** Fractions obtained by partitioning extracts from the aerial parts of *Miconia ligustroides* (MLEB) and from the flowers of *Miconia auricoma* (LAEB).

Extract	Solvent	Solvent Volume	Fraction Code	Mass (g)
MLEB (32.27 g)	Hexane	6 × 200 mL	MLEx	2.52
	Chloroform	8 × 200 mL	MLCHl_3_	5.13
	Ethyl acetate	6 × 200 mL	MLAcOEt	6.60
	H_2_O:MeOH	-	MLHM	17.10
LAEB (37.30 g)	Hexane	5 × 200 mL	LAHex	2.08
	Chloroform	7 × 200 mL	LACHl_3_	2.44
	Ethyl acetate	7 × 200 mL	LAAcOEt	7.05
	H_2_O:MeOH	-	LAHM	25.53

## Data Availability

Not applicable.

## Sample Availability

Samples of the compounds myricetin and quercetin are available from the authors.

## References

[1] Borges L.P., Amorim V.A. (2020). Secondary Plant Metabolites. Rev. Agrotecnol..

[2] Bruce S.O., Onyegbule F.A., Zepka L.Q., Nascimento T.C., Jacob-Lopes E. (2021). Biosynthesis of Natural Products. Bioactive Compounds—Biosynthesis, Characterization and Applications.

[3] Weston L.A., Mathesius U. (2013). Flavonoids: Their Structure, Biosynthesis and Role in the Rhizosphere, Including Allelopathy. J. Chem. Ecol..

[4] Khalid M., Saeed-ur-Rahman, Bilal M., Dan-feng H. (2019). Role of flavonoids in plant interactions with the environment and against human pathogens—A review. J. Integr. Agric..

[5] Buer C.S., Imin N., Djordjevic M.A. (2010). Flavonoids: New Roles for Old Molecules. J. Integr. Plant Biol..

[6] Brunetti C., Ferdinando M.D., Fini A., Pollastri S., Tattini M. (2013). Flavonoids as Antioxidants and Developmental Regulators: Relative Significance in Plants and Humans. Int. J. Mol. Sci..

[7] Valli M., Russo H.M., Bolzani V.S. (2018). The potential contribution of the natural products from Brazilian biodiversity to bioeconomy. An. Acad. Bras. Ciênc..

[8] Carvalho L.B., Cruz-Hipolito H., González-Torralva F., Alves P.L.C.A., Christoffoleti P.J., Prado R. (2011). Detection of sourgrass (*Digitaria insularis*) biotypes resistant to glyphosate in Brazil. Weed Sci..

[9] Pazuch D., Trezzi M.M., Guimarães A.C.D., Barancelli M.V.J., Pasini R., Vidal R.A. (2017). Evolution of natural resistance to glyphosate in morning glory populations. Planta Daninha.

[10] Silva A.F., Concenço G., Aspiazú I., Galon L., Ferreira E.A., Oliveira M.F., Brighenti A.M. (2018). Métodos de controle de planta daninhas. Controle de Plantas Daninhas: Métodos Físico, Mecânico, Cultural, Biológico e Alelopatia.

[11] Grazziero D.L.P., Lollato R.P., Brighenti A.M., Pitelli R.A., Voll E. (2015). Manual de Identificação de Plantas Daninhas da Cultura da Soja.

[12] Duke S. (2015). Proving Allelopathy in Crop–Weed Interactions. Weed Sci..

[13] Macías F.A., Galindo J.L.G., Galindo J.C.G. (2007). Evolution and current status of ecological. Phytochemistry.

[14] Saxena M., Saxena J., Nema R., Singh D., Gupta A. (2013). Phytochemistry of Medicinal Plants. J. Pharmacogn. Phytochem..

[15] Barros V.M.D.S., Pedrosa J.L.F., Gonçalves D.R., Medeiros F.C.L., Carvalho G.R., Gonçalves A.H., Teixeira P.V.V.Q. (2020). Herbicides of biological origin: A review. J. Hortic. Sci. Biotechnol..

[16] Andrade A.L.P., Moro R.S., Kuniyoshi Y.S., Carmo M.R.B. (2015). Floristic survey of the Furnas Gêmeas region, Campos Gerais National Park, Paraná state, southern Brazil. Check List..

[17] Maack R. (1950). Mapa Fitogeográfico do Estado do Paraná.

[18] Martins T.D., Vieira B.C. (2014). Os campos gerais do paraná e a contribuição da geomorfologia climática. RDG USP.

[19] Marques R.V., Ferreira Q.I.X., Alcântara C.B., Castro G.G. (2019). Estratégias de dispersão e ornitocoria em Melastomataceae em três fragmentos do cerrado. Revista de Educação, Saúde e Meio Ambiente.

[20] Moraes D.A., Cavalin P.O., Moro R.S., Oliveira R.A.C., Carmo M.R.B., Marques M.C.M. (2015). Edaphic filters and the functional strucuture of plant assemblages in grasslands in Southern Brazil. J. Veg. Sci..

[21] Rosa M.C., Moro R.S. (2016). Convergências no padrão de distribuição de espécies vegetais campestres nos Campos Gerais (Província Biogeográfica Paranaense). TerraPlural.

[22] Maia F.R., Telles F.J., Goldenberg R. (2018). Time and space affect reproductive biology and phenology in *Tibouchina hatschbachii* (Melastomataceae), an endemic shrub from subtropical grasslands of southern Brazil. J. Linn. Soc. Bot..

[23] Goldenberg R., Baumgratz J.F.A., Michelangeli F.A., Guimarães P.J.F., Romero R., Versiane A.F.A., Fidanza K., Völtz R.R., Silva D.N., Lima L.F.G. Melastomataceae in Flora e Funga do Brasil. Jardim Botânico do Rio de Janeiro. http://reflora.jbrj.gov.br/reflora/floradobrasil/FB161.

[24] Cunha G.O.S., Cruz C.D., Menezes A.C.S. (2019). An Overview of Miconia genus: Chemical Constituents and Biological Activities. Pharmacogn. Rev..

[25] Gorla C.M., Perez S.C.J.G.A. (1997). Influência de extratos aquosos de folhas de *Miconia albicans* Triana*, Lantana camara* L, *Leucaena leucocephala* (Lam) de Wit Drimys winteri Forst, na germinação e crescimento inicial de sementes de tomate e pepino. Rev. Bras. Sementes.

[26] Isaza J.H., Qi F.J.J., Usma J.L.G., Restrepo J.C. (2007). Actividad alelopática de algunas especies de los géneros *Miconia*, *Tibouchina*, *Henriettella*, *Tococa*, *Aciotis* y *Bellucia* (Melastomataceae). Sci. Fides..

[27] González F.J.J., Torres P.E. (2012). Allelopathic activity of chloroform extract from *Henriettella trachyphylla* and ethyl acetate extract from *Miconia coronata* (Melastomataceae). Sci. Fides..

[28] Morikawa C.I.O., Miyaura R., Figueroa M.L.T., Salgado E.L.R., Fujii Y. (2012). Screening of 170 Peruvian plant species for allelopathic activity by using the Sandwich Method. Weed Biol. Manag..

[29] Orbe P., Tuesta G., Merino C., Rengifo E., Cabanillas B. (2013). Evaluación de la actividad alelopática de cinco especies vegetales amazónicas. Folia Amazón..

[30] Gatti A.B., Takao L.K., Pereira V.C., Ferreira A.G., Lima M.I.S., Gualtieri S.C.J. (2014). Seasonality effect on the allelopathy of cerrado species. Braz. J. Biol..

[31] Pinto G.F.S., Kolb R.M. (2016). Seasonality affects phytotoxic potential of five native species of Neotropical savana. Botany.

[32] Santos M.A.F., Silva M.A.P., Santos A.C.B., Alencar S.R., Torquato I.H.S., Andrade A., Costs N.C., Generino M.E.M., Landim H.S., Oliveira A.H. (2015). Allelopathy of *Miconia* spp. (Melastomataceae) in *Lactuca sativa* L. (Asteraceae). J. Agric. Sci..

[33] Sotero V., Suarez P., Vela J.E., Sotero D.G., Fujii Y. (2016). Allelochemicals of Three Amazon Plants Identified by GC-MS. IJEAS.

[34] Santos M.A.F., Silva M.A.P., Santos A.C.B., Bezerra J.W.A., Alencar S.R., Barbosa E.A. (2017). Atividades biológicas de *Miconia* spp. Ruiz & Pavon (Melastomataceae Juss.). Gaia Sci..

[35] Bianchin M. (2022). Contribuições ao Estudo Fitoquímico e Atividades Biológicas de *Miconia ligustroides* e *Miconia auricoma* (Melastomataceae). Ph.D. Thesis.

[36] Rice E.L. (1964). Allelophaty.

[37] Souza-Filho A.P.S., Fonseca M.L., Arruda M.S.P. (2005). Substâncias químicas com atividades alelopáticas presentes nas folhas de *Parkia pendula* (Leguminosae). Planta Daninha.

[38] Semwal D.K., Semwal R.B., Combrinck S., Viljoen A. (2016). Myricetin: A Dietary Molecule with Diverse Biological Activities. Nutrients.

[39] Zhao L., Wang H., Du X. (2021). The therapeutic use of quercetin in ophthalmology: Recent applications. Biomed. Pharmacother..

[40] Song X., Tan L., Wang M., Ren C., Gou C., Yang B., Ren Y., Cao Z., Li Y., Pei J. (2021). Myricetin: A review of the most recent research. Biomed. Pharmacother..

[41] Cristiane R.R., Braga F.R.B., Pinto L.H.R., Mário G.C. (2015). Phytotoxic effects of phenolic compounds on *Calopogonium mucunoides* (Fabaceae) roots. Aust. J. Bot..

[42] Jacobs M., Rubery P.H. (1988). Naturally Occurring Auxin Transport Regulators. Science.

[43] Shah A., Smith D.L. (2020). Flavonoids in Agriculture: Chemistry and Roles in, Biotic and Abiotic Stress Responses, and Microbial Associations. Agronomy.

[44] Mierziak J., Kostyn K., Kulma A. (2014). Flavonoids as Important Molecules of Plant Interactions with the Environment. Molecules.

[45] Tauchen J., Huml L., Rimpelova S., Jurasek M. (2020). Flavonoids and Related Members of the Aromatic Polyketide Group in Human Health and Disease: Do They Really Work?. Molecules.

[46] Delgado M.E., Haza A.I., Arranz N., García A., Morales P. (2008). Dietary polyphenols protect against N-nitrosamines and benzo(a)pyrene-induced DNA damage (strand breaks and oxidized purines/pyrimidines) in HepG2 human hepatoma cells. Eur. J. Nutr..

[47] Hobbs C.A., Swarts C., Maronpot R., Davis J., Recio L., Koyanagi M., Hayashi S.M. (2015). Genotoxicity evaluation of the flavonoid, myricitrin, and its aglycone, myricetin. Food Chem. Toxicol..

[48] Agati G., Azzarello E., Pollastri S., Tattini M. (2012). Flavonoids as antioxidants in plants: Location and functional significance. Plant Sci..

[49] Woodward A.W., Bartel B. (2005). Auxin: Regulation, Action, and Interaction. Ann. Bot..

[50] Franco D.M., Silva E.M., Saldanha L.L., Adachi S.A., Schley T.R., Rodrigues T.M., Dokkedal A.L., Nogueira F.T., Almeida L.F.R. (2015). Flavonoids modify root growth and modulate expression of SHORT-ROOT and HD-ZIP III. J. Plant Physiol..

[51] Zhao Y. (2010). Auxin Biosynthesis and Its Role in Plant Development. Auxin Biosynthesis and Its Role in Plant Development. Annu. Rev. Plant Biol..

[52] Zhang W., Lu L.Y., Hu L.Y., Cao W., Sun K., Sun Q.B., Siddikee A., Shi R.H., Dai C.C. (2018). Evidence for the involvement of auxin, ethylene and ROS signaling during primary root inhibition of *Arabidopsis* by the allelochemical benzoic acid. Plant Cell Physiol..

[53] Parvez M.M., Tomita-Yokotani K., Fujii Y., Konishi T., Iwashina T. (2004). Effects of quercetin and its seven derivatives on the growth of *Arabidopsis thaliana* and *Neurospora crassa*. Biochem. Syst. Ecol..

[54] Coelho E.M.P., Barbosa M.C., Mito M.S., Mantovanelli G.C., Junior R.S.O., Ishii-Iwamoto E.L. (2017). The activity of the antioxidant defense system of the weed species *Senna obtusifolia* L. and its resistance to allelochemical stress. J. Chem. Ecol..

[55] Pergo E.M.C., Ishii-Iwamoto E.L. (2011). Changes in energy metabolism and antioxidant defense systems during seed germination of the weed species *Ipomoea triloba* L. and the responses to allelochemicals. J. Chem. Ecol..

[56] Abrahim D., Braguini W.L., Kelmer-Bracht A.M., Ishii-Iwamoto E.L. (2000). Effects of four monoterpenes on germination, primary root growth, and mitochondrial respiration of maize. J. Chem. Ecol..

[57] Kalinova J., Vrchotova N. (2009). Level of catechin, myricetin, quercetin and isoquercitrin in buckwheat (*Fagopyrum esculentum* Moench), changes of their levels during vegetation and their effect on the growth of selected weeds. J. Agric. Food Chem..

[58] Nasir H., Iqbal Z., Hiradate S., Fujii Y. (2005). Allelopathic potential of *Robinia pseudo-acacia* L.. J. Chem. Ecol..

[59] Ferreira W.N., Lacerda C.F., Costa R.C., Filho S.M. (2015). Effect of water stress on seedling growth in two species with different abundances: The importance of Stress Resistance Syndrome in seasonally dry tropical forest. Acta Bot. Bras..

[60] Huang H., Ullah F., Zhou D.X., Yi M., Zhao Y. (2019). Mechanisms of ROS regulation of plant development and stress responses. Front Plant Sci..

[61] Ferreira A.G., Borghetti F. (2004). Germinação: Do Básico ao Aplicado.

[62] Ximenez G.R., Santin S.M.O., Ignoato M.C., Souza L.A., Pastorini L.H. (2019). Phytotoxic potential of the crude extract and leaf fractions of *Machaerium hirtum* on the initial growth of *Euphorbia heterophylla* and *Ipomoea grandifolia*. Planta Daninha.

[63] Coelho L.E., Oliveira S.M., Souza L.A., Pastorini L.H. (2021). Phytotoxic effects of *Aeschynomene fluminensis* Vell. on the initial growth of weeds and cultivated plants. Res. Soc. Dev..

[64] Aslani F., Juraimi A.S., Ahmad-Hamdani M.S., Alam M.A., Hashemi F.S.G., Omar D., Hakim M.A. (2015). Phytotoxic interference of volatile organic compounds and water extracts of *Tinospora tuberculata* Beumee on growth of weeds in rice fields. S. Afr. J. Bot..

[65] Menezes P.V.M.C., Silva A.A., Mito M.S., Mantovanelli G.C., Stulp G.F., Wagner A.L., Constantin R.P., Baldoqui D.C., Gonçales Silva R., Oliveira A.A.C. (2021). Morphogenic responses and biochemical alterations induced by the cover crop *Urochloa ruziziensis* and its component protodioscin in weed species. Plant Physiol. Biochem..

[66] Silva I.F., Vieira E.A. (2019). Phytotoxic potential of *Senna occidentalis* (L.) link extracts on seed germination and oxidative stress of Ipe seedlings. Plant Biol..

[67] Araniti F., Graña E., Krasuska U., Bogatek B., Reigosa M.J., Abenavoli M.R., Sánchez-Moreiras A.M. (2016). Loss of gravitropism in farnesene-treated arabidopsis is due to microtubule malformations related to hormonal and ROS unbalance. PLoS ONE.

[68] Yan Z.Q., Tan J., Guo K., Yao L.G. (2020). Phytotoxic mechanism of allelochemical liquiritin on root growth of lettuce seedlings. Plant Signal. Behav..

[69] Quiao Y.J., Gu C.Z., Zhu H.T., Wang D., Zhang M.Y., Zhang Y.Z., Yang C.R., Zhang Y.J. (2020). Allelochemicals of *Panax notoginseng* and their effects on various plants and rhizosphere microorganisms. Plant Divers..

[70] Bhadoria P.B.S. (2011). Allelopathy: A Natural Way towards Weed Management. Am. J. Exp. Agric..

[71] Cechinel-Filho V., Yunes R.A. (1998). Estratégias para a obtenção de compostos farmacologicamente ativos a partir de plantas medicinais. Conceitos sobre modificação estrutural para otimização da atividade. Quím. Nova.

[72] Phan N.H.T., Thuan N.T.D., Duy N.V., Huong P.T.M., Cuong N.X., Nam N.H., Thanh N.V., Minh C.V. (2015). Flavonoids isolated from *Dipterocarpus obtusifolius*. Vietnam. J. Chem..

[73] Pabuprapap W., Wassanatip Y., Khetkam P., Chaichompoo W., Kunkaewom S., Senabud P., Hata J., Chokchaisiri R., Svasti S., Suksamrarn A. (2019). Quercetin analogs with high fetal hemoglobin-inducing activity. Med. Chem. Res..

[74] Pazuch D., Trezzi M.M., Diesel F., Barancelli M.V.J., Batistel S.C., Pasini R. (2015). Superação de dormência em sementes de três espécies de *Ipomoea*. Ci Rural..

[75] Azania C.A.M., Hirata A.C.S., Azania A.A.P.M. (2011). Biologia e manejo químico de corda-de-viola em cana-de-açúcar. Bol. Técnico IAC.

